# Incorporation of Ethylcellulose Microparticles Containing a Model Drug with a Bitter Taste into Nanofibrous Mats by the Electrospinning Technique—Preliminary Studies

**DOI:** 10.3390/ma15155286

**Published:** 2022-07-31

**Authors:** Katarzyna Olechno, Nina Katarina Grilc, Špela Zupančič, Katarzyna Winnicka

**Affiliations:** 1 Department of Pharmaceutical Technology, Medical University of Bialystok, Mickiewicza 2c, 15-222 Bialystok, Poland; 2 Department of Pharmaceutical Technology, University of Ljubljana, Aškerčeva c. 7, 1000 Ljubljana, Slovenia; nina.katarina.grilc@ffa.uni-lj.si (N.K.G.); spela.zupancic@ffa.uni-lj.si (Š.Z.)

**Keywords:** electrospinning, electrospun nanofibers, drug delivery platform, ethylcellulose, microparticles, rupatadine fumarate, polymeric materials, orodispersible films

## Abstract

Electrospinning is considered a simple and comprehensive technique to formulate ultrafine fibres by using an electric field. Polymeric nanofibers constitute promising materials in biomedical applications as drug delivery systems. For their preparation, both natural and synthetic polymers are utilised. Owing to the potential use of electrospun nanofibers as an orodispersible drug dosage form, ethylcellulose microparticles containing the antihistamine drug rupatadine fumarate, prepared by the spray drying technique to conceal the drug’s bitter taste, were incorporated into nanofibers. The obtained nanofibrous mats were evaluated for morphology, mechanical strength, disintegration time, the drug solid state and acceptability in terms of taste masking efficiency. Preliminary studies showed that hypromellose used as a single polymer was not a suitable substance for the manufacturing of nanofibers. Therefore, in order to facilitate the obtention of homogeneous nonwovens, different grades of polyethylene oxide (2,000,000–2M-Da and 4,000,000–4M-Da) were added, which improved the quality of the prepared mats. Nanofibers of the most satisfactory quality were obtained from hypromellose (6.5% *w*/*v*) and PEO (2M, 0.5% *w*/*v*). SEM image analysis has shown that the nanofibers were homogeneous and smooth and possessed a fast disintegration time (below 30 s) and an adequate drug content with a simultaneous taste-masking effect (as indicated by the in vivo and in vitro methods). However, further studies are necessary to refine their mechanical characteristics.

## 1. Introduction

In recent years, electrospun nanofibers have increasingly gained attention due to many advantages, e.g., a high drug loading capacity and encapsulation efficiency, porosity, and a small pore size and nanometer diameters. They are also characterised by a high surface area per unit mass ratio and good mechanical properties and elasticity, while retaining a fast disintegration time. Electrospinning presents a useful approach to the development of new functional nanomaterials for applications in different sectors (pharmaceutical, medical, cosmetic, etc.) [1,2]. This process involves stretching the fibers from a stream of polymeric solutions at a relevant speed through a spinning nozzle. The factor that determines the formation of fibers from a solution jet is the physical entanglement of molecules (chain entanglement) [1,2,3]. The number of molecule entanglements increases with the increase in the molecular weight of the polymer and its concentration in the solution, resulting in an increase in the viscosity of the solution, which leads to the formation of a coherent solution stream. The formed fibers usually have a circular cross-section; however, due to different rates of solvent evaporation from the solution stream, other fibers shapes such as ribbons can be formed [1,2,4]. 

The acceptability of medicinal products is a pivotal issue to ensuring adherence and therapeutic outcomes. Pharmaceutical technology is focusing on the development of patient-centric drug dosage forms that tailor to the needs of all age groups; hence, special attention is given to the orodispersible formulations, e.g., orodispersible films (ODFs). ODFs constitute a valuable dosage form for paediatric and geriatric patients or those suffering from dysphagia (swallowing difficulties), as they minimise the chocking risk and they do not need to be washed down with water [5,6,7]. Their production is carried out, among other techniques, by the electrospinning technique [8,9,10,11,12,13,14,15]. The ODFs obtained by this method possess a high surface area and good mechanical properties and elasticity, while maintaining a fast disintegration time [1,2,4]. The choice of the polymer used in the manufacturing of electrospun mats must be considered, as biomedical applications require biocompatible and biodegradable nanofibers that will not promote adverse responses when used by patients. Hydroxypropyl methylcellulose (hypromellose, HPMC) is a semisynthetic polymer produced by the chemical modification of the naturally occurring cellulose extensively utilised in pharmaceutical technology. It is a water-soluble hydrophilic non-ionic cellulose ether. Its classification as GRAS (Generally Recognised as Safe) by the Food and Drug Administration (FDA) is a reason why HPMC can be safely used in humans, including the paediatric population. This polymer rapidly disintegrates/dissolves in the oral cavity during contact with saliva, which is a desirable feature for orodispersible formulations [16,17,18,19,20,21,22]. 

This study aimed to assess the possibility of obtaining HPMC-based homogenous nanofibers, by using the electrospinning method, as carriers of the antihistamine drug rupatadine fumarate (RUP) for their potential utilisation as an orodispersible delivery platform. Because of an extremely bitter taste, RUP was chosen as a model drug. RUP is one of the newest antihistamines with a multidirectional effect; it is used both in children and adults [23]. As the designed fibers are potentially intended to serve as an oral, fast-dissolving drug carrier, taste is an important factor affecting their acceptability. Owing to the immediate exposure of orodispersible drug dosage forms to the taste buds, drug taste masking is an essential aspect of the therapeutic process’s effectiveness. Obtaining microparticles (MP) is considered one of the most effective and efficient ways of taste masking [24,25]. MPs constitute the basis for creating various formulations, including orodispersible oral forms. One of the drug taste- and odor-neutralising polymers commonly used in MP preparation is ethylcellulose (EC). EC is hydrophobic polymer considered as safe and included in the database of excipients approved for use in medicines by the FDA [17,18,19]. It creates a barrier that limits the contact with taste buds, providing a taste-masking effect. During preliminary studies, the MPs prepared using EC water dispersions (Surelease^®^—Colorcon Inc., Harleysville, PA, USA; Aquacoat^®^ ECD—FMC BioPolymer, Newark, NJ, USA) were selected for research due to the highest degree of bitterness reduction (evaluated using three various techniques: a human taste panel and in vitro methods (the electronic tongue and the drug release)) [26]. Designed HPMC-based nanofibers containing MPs were evaluated with respect to morphology, drug content, RUP solid state (by differential scanning calorimetry—DSC), and taste acceptability (accessed in vivo in human volunteers and based on the RUP release test).

## 2. Materials and Methods

### 2.1. Materials

Aquacoat^®^ ECD, an aqueous dispersion containing ethylcellulose (24.5–29.5%), a surfactant (SLS—sodium lauryl sulphate; 0.9–1.7%), and an emulsion stabiliser (cetyl alcohol; 1.7–3.3%) were obtained from FMC BioPolymer, Newark, NJ, USA. Surelease^®^ E-7-19040 (containing oleic acid—1.9%, ethylcellulose—25%, and fractionated coconut oil) was obtained from Colorcon Inc., Harleysville, PA, USA. HPMC (molecular weight 1260, Pharmacoat^®^ 606) was donated from Shin-Etsu Chemical Co., Ltd., Tokyo, Japan. Polyethylene oxide (PEO, Sigma-Aldrich, USA) with a molecular weight of 2,000,000 (2M) and 4,000,000 (4M) was obtained from Sigma-Aldrich, St. Louis, MO, USA. RUP with a purity of ~99% was obtained from Xi’An Kerui Biotechnology Co., Ltd., Xi’an, China.

### 2.2. Formulation of Electrospun Mats with MP Containing RUP

MPs were prepared by the spray drying technique using a Mini Spray Dryer B-290 (Büchi, Switzerland) provided with a conventional 0.7 mm nozzle, using Surelease^®^ (MP-S-RUP) or Aquacoat^®^ ECD (MP-A-RUP) diluted with purified water to the desired concentration and blended with RUP. An efficient bitterness-reducing barrier was formed with an RUP:polymer ratio (0.5:1) at 6% EC concentration [26]. The following spray-drying process parameters were determined experimentally: inlet temperature—85 °C, outlet temperature—70 °C, aspirator flow rate—98%, flow rate of the dispersion—3.5 mL/min. All experiments were conducted under ambient conditions: room temperature: 20–25 °C, relative humidity—RH 40–50%.

The initial electrospinning experiments revealed that HPMC alone can not be considered as a good nanofiber-forming polymer. Different concentrations—3.5, 6.5, 9.5, and 12.5% (*w*/*v*)—of HPMC were tested in the experiment. Nevertheless, these variations did not result in an improved electrospinability and mats quality. Therefore, the addition of different grades of PEO 2M or 4M at a concentration of 0.5–1% (*w*/*v*) was tested as a method to facilitate the fabrication of the uniform nanofibers. The of the most satisfying quality were obtained with an HPMC at 6.5% and a PEO 2M at 0.5%. Interestingly, only the solution containing MP-A-RUP was selected for further studies, as those containing MP-S-RUP disrupted the electrospinning process. Regardless of the used voltage, the flow rate, and the distance from the needle to the collector, stable flux was not obtained. 

Three formulations with the following compositions were prepared for the present study: placebo solution containing only HPMC and PEO (F1) which were dissolved in the ratio of 13:1; a dispersion containing RUP in a powder form (HPMC, PEO, RUP—F2) where the mass ratio between ingredients was 13:1:2, respectively; and a dispersion with RUP enclosed in the microparticles—MP-A-RUP (HPMC, PEO, MP-A-RUP—F3) where the mass ratio was 13:1:6, respectively). Distilled water was used as a solvent. The formulations were prepared at room temperature under stirring for 24 h to ensure their homogeneity. The electrospinning process was conducted using Fluidnatek^®^ LE-100 (Bioinicia, Paterna, Spain) with a vertical setup at 25 °C and 16% relative humidity (RH). The voltage was maintained at 15 kV, and the nanofibers were deposited on aluminum foil placed on a rotating drum collector rotating at 100 rpm and placed at a distance of 25 cm from the needle tip. 

### 2.3. Preliminary Evaluation of the Obtained Nanofibrous Mats 

#### 2.3.1. RUP Content 

The amount of RUP in the nanofibrous mats was determined (n = 3) by dissolving the exact amount of mat in a buffer consisting of water and NaH_2_PO_4_ (at pH = 3.0). The analysis was performed by utilising the HPLC apparatus (Agilent Technologies 1200, Santa Clara, CA, USA) using the Waters Spherisorb^®^ (Waters Corporation, Milford, CT, USA) 5 μm ODS1 4.6 × 250 mm column (Waters Corporation, Milford, CT, USA). Isocratic elution was performed by using methanol:phosphate buffer, pH = 3.0 (35:65, *v*/*v*). The flux was set at 1.0 mL/min, and the wavelength was set at 245 nm [27,28,29]. The correlation coefficient r was 0.999. The studies were carried out in triplicate.

#### 2.3.2. Mechanical Properties

Mats were tested in terms of tensile strength—TS, Young’s modulus—E, the percent of elongation at break—EB, and tear resistance—TR by a texture analyser (TA.XT plus, Stable Micro Systems, Godalming, UK). A piece of mat was placed in the bracket at a height of 20 mm and then extended with a 1 mm/s speed and a 5 kg cell loading [30]. Each study was performed at least in triplicate.

#### 2.3.3. Disintegration Time Assessment 

##### In Vivo in Healthy Volunteers

The time needed for nanofibrous mats disintegration was evaluated in the oral cavity involving six healthy volunteers (Approval of Bioethics Commission, number APK.002.112.2021). A single excerpt of mat was held in the oral cavity until mat disintegration, and then it was spat out. Purified water was used to rinse the mouth. The time needed for the total disintegration of the mat in the oral cavity was noted. The test was performed in triplicate.

##### In a Petri Dish

A single excerpt of mat was placed in a Petri dish, gently shook, and observed until complete disintegration. As a medium, 3 mL of phosphate buffer (pH 6.8) at 37 °C was used. The test was performed in triplicate.

#### 2.3.4. Differential Scanning Calorimetry (DSC)

All the substances used (individually, their physical mixtures) and the obtained nanofibers were assessed by DSC (DSC 1 STARe, Mettler Toledo, Greifensee, Switzerland). Approximately 7 mg of the samples was weighed in an aluminum pan and then tightly closed with an aluminum lid containing a pin-hole. Tests were performed in the temperature range of 25–300 °C with a heating rate of 10 °C/min and a nitrogen flow of 50 mL/min, with an empty pan constituting the reference. 

#### 2.3.5. Scanning Electron Microscopy (SEM) Imaging

To assess the morphology, the nanofibers were examined with a scanning electron microscope (SEM, Supra35 VP, Carl Zeiss, Jena, Germany) under magnification (5000×). The samples were placed on adhesive tapes fixed to the surface of a stand and evaluated without sputter-coating at the accelerated voltage of 1 kV using a secondary detector. Nanofiber diameters were measured using the Image J software (National Institutes of Health, Bethesda, MD, USA), and the average nanofiber diameters were determined as the mean values of 70 measurements of randomly selected fibers.

### 2.4. Evaluation of Taste-Masking Effectiveness

#### 2.4.1. In Vivo

The evaluation of the taste-masking efficacy of the obtained nanofibrous mats was performed in vivo, involving six healthy volunteers [30] (Approval of Bioethics Commission number APK.002.112.2021). Briefly, after the mouth was rinsed with purified water, the mat was held in the mouth for 30 s and spat out, and then the mouth was rinsed again. The volunteers used a scale with the following values: 0—not bitter; 1—slight bitter, 2—moderately bitter, and 3—very bitter. Each formulation was analysed in triplicate.

#### 2.4.2. RUP Dissolution

RUP dissolution was performed with a mini paddle and mini vessel (200 mL volume) apparatus provided with enhancer cells (Erweka Dissolution Tester DT 600HH, Heusenstamm, Germany) [26] dipped in 40 mL of phosphate buffer (pH 6.8) at 75 rpm and 37 °C (+/−0.5). A single excerpt of mat was put in a single cell and covered with membrane from an ultra-thin cellulose to prevent flotation. The amount of dissolved RUP was assessed according to Section 2.3.1 [26]. The test was performed in triplicate.

## 3. Results and Discussion

Preliminary studies have been carried out to assess the feasibility of using HPMC and EC microparticles containing the antihistamine drug rupatadine fumarate (designed to mask the taste of bitter drugs) in the electrospinning process to produce nanofibrous orodispersible mats. In the first stage of the research, the electrospinning process parameters and composition of the solution were established. The viscosity value of the spinning solutions affects the morphology of the resulting fibers. Particularly, the viscosity determines the degree of entanglement of the chain of polymer molecules in the solution: for high-viscosity liquids, the electrical charges will not create sufficient force to form fibers, and for low-viscosity liquids, the electrospinning flux might disintegrate without forming fibers. By gradually increasing the concentration of HPMC, fibers with different morphologies were obtained. For the concentration of 12.5%, the solution was too viscous, resulting in the formation of clumped fibers. For further studies, an intermediate value of 6.5% was selected (from 3.5, 6.5, 9.5, and 12.5%). However, it has been demonstrated that HPMC used as a single polymer is not suitable for the production of nanofibers with the desired morphology; they were tangled with “beads” present on their surface. It should be emphasised that the inability of some polymers to electrospin independently can be overcome by carrier use. In this work, HPMC was mixed with PEO to facilitate the fabrication of nanofibrous mats. PEO serves as a process aid to improve the electrospinning of the HPMC solution—its incorporation enhances spinnability. An addition of PEO impacts the formation of hydrogen bonds with HPMC, lowers the tension between its chains, enhancing their entanglement, and affects electrical conductivity [31]. Therefore, to improve the quality of the prepared fibers, different types of PEO with a molecular weight of 2M-Da or 4M-Da at a concentration of 0.5–1.0% (*w*/*v*) were added. The fibers obtained from the solution containing HPMC at 6.5% (*w/v*) and PEO 2M-Da at a 0.5% (*w/v*) concentration were characterised by a satisfactory morphology, and they were smooth and devoid of spindles and beads.

**Figure 1:**
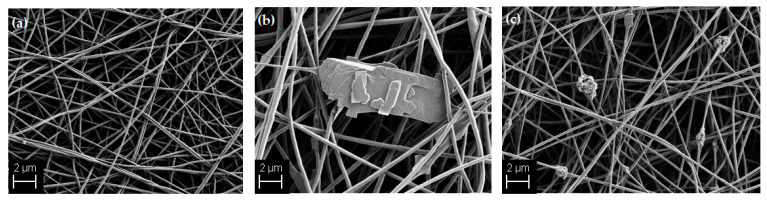
SEM images of the obtained nanofibers (magnification 5000×) of (**a**) placebo nanofibers (F1); (**b**) nanofibers with RUP in powder form (F2); (**c**) nanofibers with MP-A-RUP incorporated (F3). Incorporated microparticles with RUP (MP-A-RUP) are observed as small microporous particles along with electrospun fibers.

The analysis of the SEM images (Figure 1) revealed that the obtained nanofibers were uniform, smooth, well-shaped, and devoid of beads. The placebo nanofibers are in Figure 1a; those with pure RUP in powder (crystalline) form are in Figure 1b; and those with incorporated MP-A- RUP are in Figure 1c. The average diameter of the placebo nanofibers nanofibres was 340.21 ± 31.16 nm; that of the RUP-loaded nanofiebrs was 356.81 ± 41.06 nm; and that of the MP-A RUP-loaded nanofibers was 406.20 ± 37.96 nm.

**Figure 2:**
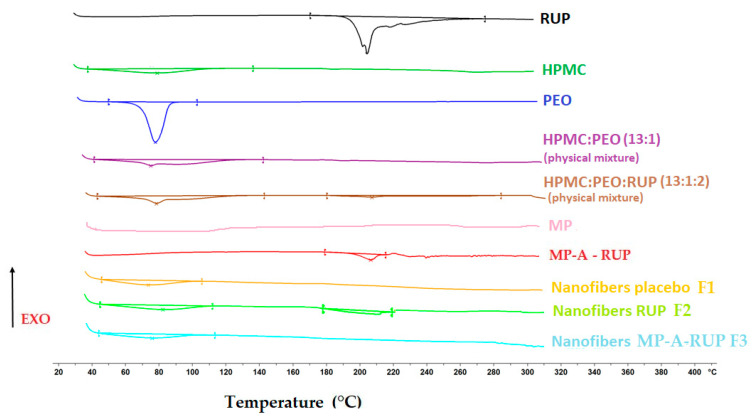
DSC thermograms of pure RUP; HPMC; PEO; physical mixtures of HPMC:PEO (13:1) and HPMC:PEO:RUP (13:1:2); MP; MP-A-RUP; and the obtained nanofibers (placebo (F1), those with RUP in a powder form (F2), and those with MP-A-RUP (F3).

DSC tests were conducted to assess the physical drug characteristics and the possible incompatibilities (Figure 2). The thermogram of the pure RUP exhibited an endothermic event at 200.35°C, marked by a sharp peak which corresponded to its melting point [32,33]. The thermogram of the MP placebo shows no thermal event, revealing that the aqueous EC dispersion used was in an amorphous state. The transformation of RUP into MP did not change the nature of the drug in the solid state, but the peak shifted to 195.78 °C. In the physical mixture of HPMC, PEO, and RUP, there was a small peak which corresponded to the melting point of RUP. The endothermic peak with similar melting enthalpy was also observed in case of nanofibers with RUP, which means that the RUP crystallinity was not changed during electrospinning. This is in accordance with the SEM image (Figure 1), where crystals of RUP are seen incorporated into nanofibers. However, in DSC curves for the nanofibers with MP-A-RUP no melting peak of RUP was detected. This lack of a RUP melting peak likely arose from the presence of crystalline RUP in undetectable concentration. 

As the mats were designed to serve as orodispersible films, for which mechanical properties are of the most importance, the requirements for this dosage form must be taken into account during their evaluation. However, the pharmacopoeias do not provide guidance while referring to the mechanical features of orodispersible films. European Pharmacopoeia (Ph. Eur.) only indicates that the films “should possess suitable mechanical strength to resist handling without being damaged”, without guidance on the test methods [34,35]. The methods of orodispersible films analysis are described in scientific publications [36,37,38,39,40,41], or analyses are carried out based on the standards used to assess the quality of plastics [42,43,44,45,46]. Mechanical properties are crucial factors, as they supply the data on the mechanical strength and the ductility of films, parameters relevant during packing, transport, and application [47]. Materials possessing higher values of tensile strength and a higher Young’s modulus are usually hard and brittle. Films that are too flexible might be susceptible to deformation, and brittle formulations might crack during production and cutting. Formulations with optimal mechanical properties should have moderate tensile strength values, low Young’s modulus values, and a high elongation percentage [36,37,38]. It should also be taken into account that the solids content has an effect on the mechanical characteristics of the obtained mats—the more solids incorporated in the matrix, the more brittle and the less flexible they are. Unfortunately, the obtained mats were characterised by poor mechanical properties—a low tear resistance and tensile strength. It can be assumed that reducing the solid quantity would improve the mat characteristics. The low values of the Young’s modulus indicate the lack of stiffness of the material. They were fragile, soft, and fluffy and therefore challenging to handle. However, the nanofiber mats were characterised by an optimal disintegration time—they disintegrated below 30 s, which confirms the possibility of their utilisation as an orodispersible dosage form. The properties of the obtained mats are shown in Table 1. 

**Figure 3:**
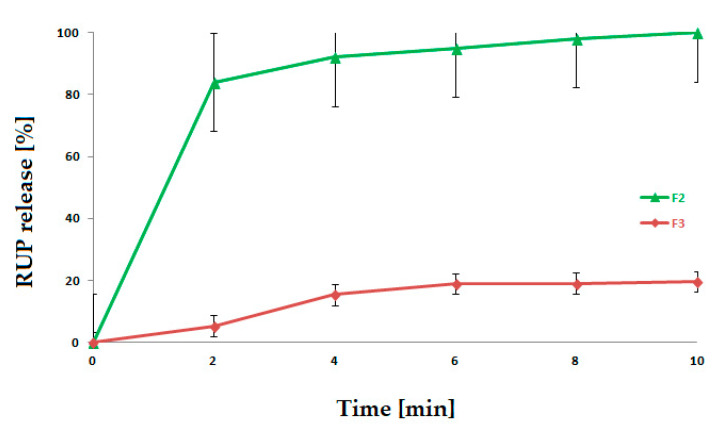
RUP dissolution from nanofibrous mats (F2, F3) evaluated in a type II apparatus.

The assessment of the drug amount indicated a homogeneous RUP content in the prepared mats (2.52 mg ± 0.33 per dosage unit). The thickness (about 15 µm) provided a dose of 2.5 mg rupatadine fumarate for the mats with area of 8 cm^2^. No drug lost during the electrospinning process was observed. The taste masking effect of the obtained formulations was determined in vivo by a human taste panel and in vitro by a drug dissolution test. The formulation prepared with MP-A-RUP (F3) was rated as non-bitter to slightly bitter (Table 2) compared to the formulation containing pure RUP (F2), which is in agreement with our previous results [26,30]. In the dissolution test, it was shown that formulation F2 released RUP very fast, whereas F3 was characterised by a much slower drug release (Figure 3). Drug dissolution is strongly correlated with the effectiveness of taste masking. The results received from the test correlated with the feedback of the volunteers. 

## 4. Conclusions

This study proved the possibility of producing nanofibers with EC microparticles containing rupatadine fumarate by using the hydrophilic polymer HPMC, but only when mixed with PEO, which significantly improved the electrospinning process. The obtained nanofibrous mats were characterised by a very fast disintegration time, and they might constitute a promising material to serve as an orodispersible drug delivery platform. However, further optimisation of the formulations’ composition, especially with regard to improving their mechanical features, is required.

## Figures and Tables

**Table 1:** Mechanical properties of obtained nanofibrous mats (*n* = 3).

Formulation	Tear Resistance [N]	Tensile Strength [N/mm^2^]	Elongation at Break [%]	Young’s Modulus [MPa]	Disintegration Time [s]
**F1**	3.14 ± 0.31	3.92 ± 0.015	5.1 ±0.03	220.0 ±0.72	<30 s
**F2**	1.67 ± 0.34	2.08 ± 0.01	5.0 ± 0.03	179.0 ± 0.58	<30 s
**F3**	1.37 ± 0.27	1.71 ± 0.06	4.0 ± 0.05	169.0 ±0.69	<30 s

**Table 2:** Organoleptic assessment of formulated ODFs: 0—without bitterness, 1—slight bitterness, 2—moderate bitterness, 3—significant bitterness.

Volunteer/ Formulation	Score		
	F1	F2	F3
A	0	3	1
B	0	2	0
C	0	3	1
D	0	3	1
E	0	2	0
F	0	3	1

## Data Availability

Data are contained within the article.
